# Low PrEP adherence despite high retention among transgender women in Brazil: the PrEParadas study

**DOI:** 10.1002/jia2.25896

**Published:** 2022-03-07

**Authors:** Emilia M. Jalil, Thiago S. Torres, Paula M. Luz, Laylla Monteiro, Ronaldo I. Moreira, Cristiane R. V. de Castro, Iuri da C. Leite, Marcello Cunha, Rita de Cássia Elias Estrela, Michelle Ramos, Brenda Hoagland, Sandra Wagner Cardoso, Peter Anderson, Valdilea G. Veloso, Erin Wilson, Beatriz Grinsztejn, for the PrEParadas Study Team

**Affiliations:** ^1^ National Institute of Infectious Diseases Evandro Chagas Fiocruz Rio de Janeiro Brazil; ^2^ Escola Nacional de Saude Publica, Fiocruz Rio de Janeiro Brazil; ^3^ Department of Pharmaceutical Sciences University of Colorado Aurora Colorado USA; ^4^ San Francisco Department of Public Health San Francisco California USA

**Keywords:** adherence, HIV, hormones, PrEP, prevention, transgender women

## Abstract

**Introduction:**

We aimed to evaluate daily oral pre‐exposure prophylaxis (PrEP) uptake, retention, and adherence and predictors of study non‐attendance and low PrEP adherence in a Brazilian trans‐specific 48‐week study (PrEParadas).

**Methods:**

We enrolled transgender women (TGW) engaging in high‐risk sexual behaviours between August 2017 and December 2018. PrEP adherence was based on tenofovir diphosphate concentrations in dried blood spots (DBS). We used random effects logistic regression models and ordinal models to estimate the odds of having a missed visit and of low PrEP adherence, respectively. Multivariable models were adjusted for variables with *p*‐value<0.10 in the univariate analysis.

**Results:**

From the 271 eligible, 130 participants were enrolled in the study (PrEP uptake: 48%), out of which 111 (85.4%) were retained at 48 weeks. Multivariable model for study non‐attendance included study visit, age, main sexual partner and stimulant use. The odds of missing a visit increased after the week 24. Participants aged 18–24 (adjusted odds ratio [aOR] = 8.76, 95% CI: 2.09–36.7) and 25–34 years (aOR = 6.79, 95% CI: 1.72–26.8) compared to TGW aged 35+ years had significantly higher odds of having a missed visit. The odds of a missed visit were higher among participants reporting stimulant use (aOR = 4.99, 95% CI: 1.37–18.1) compared to no stimulant use. DBS levels at week 48 showed that 42 (38.5%), 14 (12.8%) and 53 (48.6%) of 109 participants had low, moderate and high PrEP adherence. Multivariable model for low PrEP adherence included study visit, age, schooling, race/colour, housing, binge drinking, stimulant use, feminizing hormone therapy (FHT) use and received text message. Low PrEP adherence was significantly higher among participants with less years of schooling (aOR = 6.71, 95% CI: 1.30–34.5) and had a borderline association with Black colour/race (aOR = 6.72, 95% CI: 0.94–47.8). Participants using the FHT available at the site had decreased odds of low PrEP adherence (aOR = 0.38, 95% CI: 0.16–0.88). No participant seroconverted over the course of the study.

**Conclusions:**

Although high PrEP retention can be achieved in a gender‐affirming setting, PrEP adherence may be an important challenge faced among TGW due to social disparities. The scale‐up of prevention tools like PrEP will have to address systemic social determinants as these stand as important barriers for TGW's access to health services.

## 1 INTRODUCTION

In countries with concentrated epidemics, transgender women (TGW) bear one of the highest burdens of HIV among all population groups. HIV prevalence among TGW in low‐ and middle‐income countries (LMIC) is 48.8 times greater than that of adults of reproductive age [1]. In Brazil, despite the extraordinary HIV vulnerability, few TGW have been engaged in HIV prevention [2, 3].

Daily oral pre‐exposure prophylaxis (PrEP) with tenofovir disoproxil fumarate 300 mg (TDF) and emtricitabine 200 mg (FTC) is an efficacious and safe strategy to prevent HIV [4, 5]. However, PrEP efficacy is predicated on optimal adherence, which is a challenge for all populations, including TGW [6]. Data from the iPrEX study identified low adherence levels among TGW, but no seroconversions among those who adhered to PrEP [7]. A downward trend in drug levels over time was observed in the PrEP Brasil study, the first Brazilian PrEP demonstration study, though the small number of TGW hindered further evaluation of the underlying reasons for this finding [5]. Although PrEP has been widely available in the Brazilian public health system, data on PrEP effectiveness among TGW in Brazil are limited.

Little prior research has examined PrEP retention among TGW, limiting the implementation of HIV prevention trials that seek to meaningfully include or focus entirely on TGW. Several authors called attention to how TGW were often aggregated with men who have sex with men (MSM) in intervention trials, which belied their HIV risk and implied that methods to reach and engage TGW were the same [8]. TGW represented only 14% of the iPrEX study sample, and demographics and behavioural factors were significantly different between TGW and MSM [7]. However, iPrEX was not designed to meet the needs of TGW, perhaps explaining the low adherence observed among TGW, yet no data on differences in PrEP retention were reported. Studies increasingly highlight the differences between TGW and MSM populations and the need to conduct trans‐specific PrEP research [9], particularly addressing facilitators and barriers, including transphobia and concomitant use of feminizing hormone therapy (FHT).

Despite the urgent need, few PrEP studies were specifically designed for TGW worldwide, especially in LMIC. To address this gap, we have designed and implemented the PrEParadas, a 48‐week demonstration study to assess the feasibility of daily oral TDF/FTC provided at no cost to TGW engaging in HIV high‐risk behaviour. This analysis aimed to evaluate PrEP uptake, retention and adherence as well as factors associated with study non‐attendance and low PrEP adherence.

## 2 METHODS

### 2.1 Study population and design

PrEParadas was conducted at the National Institute of Infectious Disease Evandro Chagas (INI)‐Fiocruz, in Rio de Janeiro, Brazil, between August 2017 and December 2019. Inclusion criteria were: (1) male sex assigned at birth, (2) self‐identification as TGW or any other gender identity of the feminine spectrum, (3) age ≥18 years, (4) negative HIV status at screening and enrolment, (5) residence in Rio de Janeiro or its metropolitan area, (6) engagement in at least one of the following (i) condomless anal or neovaginal sex in the last 6 months, (ii) current sexual partner living with HIV regardless of HIV viral load, (iii) sex work in the last 6 months or (vi) sexually transmitted infection (STI) diagnosis or self‐report in the last 12 months. TGW were excluded in case of creatinine clearance <60 mL/minute, severe medical comorbidity or antiretrovirals use. INI‐Fiocruz Institutional Review Board reviewed and approved the study. All participants provided written informed consents prior to any procedure.

### 2.2 Study procedures

Participants were recruited through peer referral, by our community education team, and from the HIV testing and post‐exposure prophylaxis services available at our site. We assessed inclusion/exclusion criteria at the pre‐screening and screening visits. After enrolment, follow‐up visits and drug refills occurred at week 4 and quarterly thereafter (weeks 12, 24, 36 and 48). All participants received risk reduction counselling and clinical and safety laboratory evaluations (including HIV testing and pooled or individual HIV viral load) at every study visit. As needed, TGW had access to mental health and endocrinological care and could receive FHT available at the site (estradiol valerate 2 mg plus spironolactone 100 mg), with doses adjusted by the study endocrinologist.

HIV testing followed the Brazilian Ministry of Health algorithm [2, 10]. Syphilis testing occurred at screening and quarterly using Venereal Disease Research Laboratory tests, and positive cases were confirmed with a microhaemagglutination assay for Treponema pallidum (WAMA Diagnóstica, São Paulo, Brazil). We screened for rectal Chlamydia trachomatis (CT) and Neisseria gonorrhoea (NG) infection at enrolment and week 48 with the Abbott Real Time platform and the Amplification Reagent Kit (Abbott Molecular, Des Plains, IL, USA). In case of indeterminate results, we repeated the tests on the same samples. Hepatitis B and C status was evaluated at enrolment and, if negative, repeated at week 48 (anti‐HBs, HBs antigen, anti‐HBc for hepatitis B and anti‐HCV for hepatitis C, respectively). In case of negative hepatitis B antigen and no previous vaccine at enrolment, participants were referred for immunization at the site. Dried blood spots (DBS) for tenofovir diphosphate (TFV‐DP) and emtricitabine triphosphate assessments were collected at every follow‐up visit. We used liquid chromatography‐mass spectrometry or mass spectrometry to measure both metabolites at the University of Colorado Antiviral Pharmacology Laboratory (Aurora, CO, USA) [11, 12, 13]. The Fiocruz Laboratory performed all other laboratory testing.

Participants answered face‐to‐face questionnaires administered by experienced interviewers to capture data on socio‐demographics, sexual behaviour, FHT use, violence, alcohol and drug use, mental health, HIV risk perception, previous HIV testing and PrEP awareness. All participants were invited to receive weekly text messages (“Are you okay?”) or not (randomization 1:1). Participants randomized to receive messages who responded to one with “Not okay” or did not answer for 3 consecutive weeks received a call from the study team.

### 2.3 Study definitions

Age was broken into categorical age ranges (“18–24,” “25–34” and “35+” years). We categorized self‐reported race/colour as “White,” “Black,” “Pardo” (which is loosely translated as mixed), “Indigenous” and “Asian.” Years of schooling was categorized for analysis into “0–7” and “8+.” We collected data on housing as “own,” “rent” and “other.” Participants had to choose between three items that best described their perceived financial situation (“I have enough money to live comfortably,” “I can barely get by on the money I have” and “I cannot get by on the money I have”). We considered the first category as a “comfortable” situation and the last two options as “less than comfortable.” Sex work was defined as sex in exchange for money, drugs, gifts or favours. TGW on any estrogen‐containing regimen were defined as using FHT. Participants who used FHT provided by the study, even if non‐exclusively, were considered as using the FHT available at the site.

Syphilis infections were defined as incident if there was no previous infection reported or if the participant had a four times increase in titre (and titre was ≥1:8) after adequate treatment for prior diagnosis. Any STI diagnosis aggregated syphilis, CT or NG diagnosis at the visit that this occurred. Participants who responded affirmatively to “How often did you have 6+ drinks on one occasion in the last 3 months?” were considered positive for binge drinking [14]. Stimulant use was considered use of any of the following in the last 3 months: cocaine (powder, crack or paste), amphetamines or club drugs (ecstasy, LSD, GHB and ketamine). We used the Patient Health Questionnaire‐2 to screen for depression and considered a score of at least 3 as a positive result [15, 16]. HIV risk perception was assessed with the question “What is your chance of getting HIV in the next year?” with five possible options ranging from zero to 100%, grouped afterwards into two categories: <75% and 75%+.

### 2.4 Outcomes

We calculated PrEP uptake in the study as the percentage of enrolled participants divided by the potentially eligible. Study visit attendance (Y/N) was evaluated at each study follow‐up visit. PrEP adherence was based on TFV‐DP concentrations in DBS and categorized into: low (<350 fmol/punch, suggestive of <2 doses of TDF/FTC per week), moderate (350–699 fmol/punch, suggestive of 2–3 doses of TDF/FTC per week) and high (≥700 fmol/punch, suggestive of ≥4 doses of TDF/FTC per week) [17]. Lastly, TFV‐DP concentrations of ≥1250 fmol/punch were suggestive of daily dosing [11]. We defined PrEP retention as attendance to the week 48 visit.

### 2.5 Statistical analysis

Descriptive analysis used medians and interquartile ranges for numeric variables and absolute numbers and proportions for categorical variables. Asian and Indigenous people were excluded from the multivariate analyses because any findings regarding race for these groups may not be valid due to the small sample size. We used chi‐squared or Fisher exact tests for comparisons, as appropriate. We also provide the distribution of participants by DBS levels over the study visits. Finally, we evaluated PrEP adherence measures of each participant by visit pairs (i.e. week 12 with 4, week 24 with 12, week 36 with 24 and week 48 with 36) to calculate the transition probability of PrEP adherence level from one visit to the following.

For study visit attendance, we performed logistic regression with random effects to estimate the odds of missing a study visit. For PrEP adherence, an ordinal logistic model with random effects was used to estimate the odds of having low PrEP adherence. In both models, random effect accounts for the error correlation between repeated measures in each participant. Adjusted models included all variables with *p*‐value <0.10 in the unadjusted analyses.

We estimated incidence rates of syphilis by dividing the number of incident cases by the sum of person‐time of follow‐up. The proportions of rectal CT and NG at enrolment and week 48 were compared using generalized estimating equation logistic regression. All analyses were performed using R Software version 4.0.3.

## 3 RESULTS

**Figure 1:**
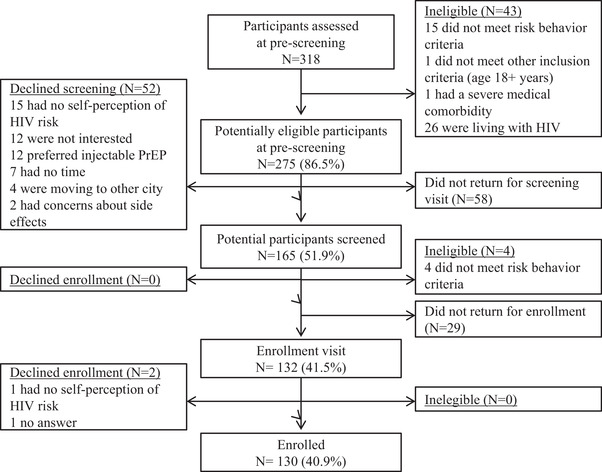
Flow‐chart of PrEParadas study (screening and enrolment assessments), 2017–2018.

Between August 2017 and December 2018, we pre‐screened 318 TGW for participation. Among 271 eligible (275 initially minus 4 additionally), 130 were enrolled in the study (PrEP uptake: 48% [130/271]) (Figure 1). Eligibility criteria were met by: 63.1% for receptive or insertive condomless anal sex in the last 6 months, 36.2% for sex work in the last 6 months, 29.2% for STI diagnosis or self‐report in the last 12 months and 3.9% for having a current sexual partner who is living with HIV. Table 1 describes participants’ characteristics at baseline.

**Table 1:** Characteristics of participants in the PrEParadas study (*N* = 130), Rio de Janeiro, Brazil, 2017–2019

	*N* (%)
Age (years)	
18–24	36 (27.69)
25–34	49 (37.69)
35+	45 (34.62)
Schooling (years)	
0–7	32 (24.62)
8–11	71 (54.62)
12+	27 (20.77)
Race/colour	
Black	35 (26.92)
*Pardo*	54 (41.54)
White	36 (27.69)
Asian or Indigenous	5 (3.85)
Housing	
Own/rent	92 (70.77)
Other^a^	38 (29.23)
Perceived financial situation	
Enough to live comfortably	44 (33.85)
Barely get by on the money I have	65 (50.00)
Cannot get by on the money I have	16 (12.31)
Missing	5 (3.85)
Main sexual partner^b^	
No	52 (40.00)
Yes	74 (56.92)
Missing	4 (3.08)
Sex work	
No	76 (58.46)
Yes	50 (38.46)
Missing	4 (3.08)
Condomless receptive anal sex	
No	45 (34.62)
Yes	80 (61.54)
Missing	5 (3.85)
Binge drinking	
No	44 (33.85)
Yes	82 (63.08)
Missing	4 (3.08)
Stimulant use	
No	106 (81.54)
Yes	20 (15.38)
Missing	4 (3.08)
Physical violence	
No	89 (68.46)
Yes	37 (28.46)
Missing	4 (3.08)
Current FHT use	
No	48 (36.92)
Yes	81 (62.31)
Missing	1 (0.77)
Ever use of fillers	
No	54 (41.5)
Yes	76 (58.5)
Positive screening depression^c^	
No	98 (75.38)
Yes	28 (21.54)
Missing	4 (3.08)
STI diagnosis^d^	
No	85 (65.38)
Yes	38 (29.23)
Missing	7 (5.38)
Self‐perceived HIV risk	
<75%	116 (89.23)
75%+	10 (7.69)
Missing	4 (3.08)
Received text message	
No	82 (63.08)
Yes	48 (36.92)

Abbreviations: FHT, feminizing hormone therapy; HIV, human immunodeficiency virus; STI, sexually transmitted infections.

^a^ Includes people living in house/apartment that belongs to the family, guest house, shelter or institution, non‐governmental organization, at work, living as a favour and homeless.

^b^ Someone that the participant considers as her primary sexual partner and feels committed to.

^c^ 3+ score in the Patient Health Questionnaire‐2.

^d^ Prevalent syphilis, rectal *Chlamydia* or *Gonorrhoea*.

Out of 130 participants enrolled, 111 (85.4%) were retained in the study at week 48. Overall, there were 84 missed visits (12 [9.2%] at week 4, 12 [9.2%] at week 12, 20 [15.0%] at week 24, 21 [16.0%] at week 36 and 19 [15.0%] at week 48, *p* = 0.05 [chi‐squared test for trend in proportions]) for an average of 12.9% across the study. The odds of missing a visit were significantly higher for participants aged 18–24 and 25–34 years compared to those ≥35 years (aOR = 8.76, 95% CI: 2.09–36.7 and aOR = 6.79, 95% CI: 1.72–26.8, respectively) (Table 2). TGW who reported stimulant use at enrolment had significantly higher odds of missing a visit (aOR = 4.99, 95% CI: 1.37–18.1).

**Table 2:** Predictors of study non‐attendance among TGW enrolled in PrEParadas study, Rio de Janeiro, Brazil, 2017–2019

	Unadjusted			Adjusted		
	OR	95% CI	*p*‐value	aOR	95% CI	*p*‐value
Study visit						
Week 4	1			1		
Week 12	1.00	0.36, 2.80	>0.9	1.04	0.38, 2.87	0.94
Week 24	2.52	0.96, 6.63	0.06	2.57	0.99, 6.63	0.05
Week 36	2.77	1.06, 7.28	**0.04**	2.83	1.10, 7.28	**0.03**
Week 48	2.28	0.86, 6.02	0.10	2.35	0.91, 6.09	0.08
Age (years)^a^						
18–24	10.6	2.36, 47.9	**0.0021**	8.76	2.09, 36.7	**0.0030**
25–34	9.01	2.18, 37.2	**0.0024**	6.79	1.72, 26.8	**0.0063**
35+	1			1		
Schooling (years)^a^						
0–7	1.77	0.43, 7.22	0.40			
8+	1					
Race/colour^a^, ^b^						
Black	1.47	0.31, 6.93	0.60			
Mixed/*Pardo*	0.62	0.15, 2.62	0.50			
White	1					
Housing^a^						
Own/rent	1					
Other^c^	2.33	0.63, 8.55	0.20			
Perceived financial situation^a^						
Comfortable	1					
Less than comfortable	1.19	0.35, 4.10	0.80			
Main sexual partner^a^, ^d^ (ref: No)	3.38	0.98, 11.6	0.05	2.32	0.77, 6.94	0.13
Sex work^a^ (ref: No)	1.89	0.57, 6.26	0.30			
Condomless receptive anal sex^a^ (ref: No)	1.78	0.51, 6.20	0.40			
Binge drinking^a^ (ref: No)	2.24	0.66, 7.58	0.20			
Stimulant use^a^ (ref: No)	7.17	1.73, 29.7	**0.0066**	4.99	1.37, 18.1	**0.02**
Physical violence (ref: No)	0.50	0.14, 1.88	0.30			
Any FHT use^a^ (ref: No)	0.60	0.17, 2.09	0.40			
Positive screening depression^a^, ^e^ (ref: No)	0.81	0.19, 3.38	0.80			
STI diagnosis^a^, ^f^	2.78	0.74, 10.4	0.13			
Self‐perceived HIV risk^a^						
<75%	1					
75%+	0.32	0.03, 3.21	0.30			
Received text message	0.62	0.18, 2.19	0.50			

Abbreviations: aOR, adjusted OR; CI, confidence interval; FHT, feminizing hormone therapy; HIV, human immunodeficiency virus; OR, odds ratio; STI, sexually transmitted infection; TGW, transgender women.

^a^ Baseline data.

^b^ Asian and Indigenous participants excluded.

^c^ Includes people living in house/apartment that belongs to the family, guest house, shelter or institution, non‐governmental organization, at work, living as a favour and homeless.

^d^ Someone that the participant considers as her primary sexual partner and feels committed to.

^e^ 3+ score in the Patient Health Questionnaire‐2.

^f^ Syphilis, rectal *Chlamydia* or *Gonorrhoea*.

Participants with a high PrEP adherence measure at the preceding visit had an 86% probability of having a similarly high PrEP adherence measure at the current visit. Also, the probability of a low PrEP adherence measure in a given visit was 82% if the preceding measure was low (Table 3). The proportion of participants who had concentrations consistent with 4+ doses/week decreased over time, from 68.8% at week 4 to 48.6% at week 48 (*p* = 0.005). DBS levels at week 48 showed that 42 (38.5%), 14 (12.8%) and 53 (48.6%) of 109 participants had low, moderate and high PrEP adherence, respectively. Only 32 (27.9%) had DBS levels suggestive of daily dosing. In the adjusted model, the odds of having a low PrEP adherence increased over time (Table 4). Low PrEP adherence was significantly higher among participants with less years of schooling (aOR = 6.71, 95% CI: 1.30–34.5) and had a borderline association with Black race/colour (aOR = 6.72, 95% CI: 0.94–47.8). Participants on the FHT available at the site had decreased odds of low PrEP adherence (aOR = 0.38, 95% CI: 0.16–0.88).

**Table 3:** Correlation between PrEP adherence by DBS measured at current and immediately prior visits among TGW enrolled in the PrEParadas study, Rio de Janeiro, Brazil, 2017–2019

DBS level observed at visit “*t*–1”	DBS level observed at visit “*t*”			Total
	Low	Moderate	High	
Low	86 (83.5%)	9 (8.7%)	8 (7.8%)	103
Moderate	20 (31.7%)	22 (34.9%)	21 (33.3%)	63
High	17 (6.4%)	32 (12.0%)	218 (81.6%)	267

Note: Low level: <350 fmol/punch, moderate: 350–699 fmol/punch and high: ≥700 fmol/punch. Table 3 shows the absolute and relative frequencies of DBS measure transitions from one visit to the following. These percentages estimate the transition probabilities Pr (Yit | Yit − 1) based on the correlation between PrEP adherence by DBS of each participant by visit pairs (i.e. a specific visit and its following visit). There are 433 transitions (or “pairs”) between the 563 observations since there is no participant's status before the first visit. Among participants with a low DSB level in the visit “*t*–1,” 83.5% maintained a low DBS level in the following visit (“*t*”). Among participants with a high DBS level in the visit “*t*–1,” 81.6% maintained a high DBS level in the next visit (“*t*”).

Abbreviations: DBS, dried blood spots; TGW, transgender women.

**Table 4:** Predictors of low PrEP adherence among TGW enrolled in PrEParadas study, Rio de Janeiro, Brazil, 2017–2019

	Univariable			Multivariable		
	OR	95% CI	*p*‐value	aOR	95% CI	*p*‐value
Study visit						
Week 4	1			1		
Week 12	1.82	0.86, 3.85	0.12	1.86	0.85, 4.09	0.12
Week 24	4.70	2.17, 10.2	**0.00009**	4.33	1.92, 9.77	**0.0004**
Week 36	3.76	1.72, 8.19	**0.0009**	3.73	1.66, 8.38	**0.0015**
Week 48	8.33	3.76, 18.4	**0.0000002**	6.79	2.79, 16.5	**0.00002**
Age (years)						
18–24	9.56	2.36, 38.70	**0.0016**	4.58	0.74, 28.5	0.10
25–34	2.03	0.56, 7.35	0.30	1.51	0.30, 7.50	0.61
35+	1			1		
Schooling (years)						
0–7	7.63	1.77, 32.9	**0.0064**	6.71	1.30, 34.5	**0.02**
8+	1			1		
Race/colour^a^						
Black	8.12	1.57, 42.0	**0.01**	6.72	0.94, 47.8	0.06
Mixed/*Pardo*	1.81	0.41, 7.99	0.40	1.81	0.33, 9.92	0.50
White	1			1		
Housing						
Own/rent	1			1		
Other^b^	1.87	1.87, 1.88	**<0.0000000000000002**	0.99	0.20, 4.83	0.99
Perceived financial situation						
Comfortable	1					
Less than comfortable	2.38	0.61, 9.27	0.20			
Main sexual partner^c^ (ref: No)	0.75	0.38, 1.48	0.40			
HIV‐positive sexual partner (ref: No)	0.32	0.04, 2.45	0.30			
Sex work (ref: No)	0.90	0.37, 2.19	0.80			
Condomless receptive anal sex (ref: No)	1.08	0.55, 2.12	0.80			
Binge drinking (ref: No)	2.27	1.17, 4.38	**0.02**	1.87	0.91, 3.83	0.09
Stimulant use (ref: No)	3.15	1.26, 7.86	**0.01**	1.87	0.68, 5.10	0.22
Physical violence (ref: No)	1.04	0.55, 1.98	0.90			
FHT use						
No	1					
Option available at the site	0.43	0.43, 0.43	**<0.0000000000000002**	0.38	0.16, 0.88	**0.03**
Other	0.68	0.68, 0.68	**<0.0000000000000002**	0.71	0.26, 1.97	0.51
Positive screening depression^d^ (ref: No)	2.24	0.52, 9.57	0.30			
Self‐perceived HIV risk						
<75%	1					
75%+	0.33	0.04, 2.70	0.30			
Received text message	0.56	0.56, 0.56	**<0.0000000000000002**	1.34	0.32, 5.60	0.69

Abbreviations: aOR, adjusted OR; CI, confidence interval; FHT, feminizing hormone therapy; HIV, human immunodeficiency virus; OR, odds ratio; PrEP, pre‐exposure prophylaxis; TGW, transgender women.

^a^ Asian and Indigenous participants excluded.

^b^ Includes people living in house/apartment that belongs to the family, guest house, shelter or institution, non‐governmental organization, at work, living as a favour and homeless.

^c^ Someone that the participant considers as her primary sexual partner and feels committed to.

^d^ 3+ score in the Patient Health Questionnaire‐2.

Overall, 80 (64.0%), 67 (63.8%) and 67 (65.0%) of participants reported condomless receptive anal sex at enrolment, 24 and 48 weeks, respectively. The mean number of condomless receptive anal sex in the last 30 days remained during the study (3.1 at enrolment and 3.74 at week 48). The mean number of sexual partners in the previous month was 8.1 (95% CI: 5.3–10.9) at enrolment and 7.9 (95% CI: 4.9–10.9) at week 48. During the study follow‐up, there was no indication of acute HIV infection; no participant seroconverted.

At enrolment, 12 (9.2%, 95% CI: 4.0–14.3) of 130 participants had CT, compared to 11 (10.1%, 95% CI: 4.4–15.8) of 109 participants at week 48 (*p* = 0.81). All participants who tested positive for CT/NG at baseline received treatment before the 48‐week visit. Prevalence of rectal NG ranged from 7.5% (9/130, 95% CI: 2.8–12.1) at enrolment to 8.3% (9/109, 95% CI: 3.1–13.4) at week 48 (*p* = 0.83). From baseline to 48 weeks, syphilis incidence was 25.8/100 person‐years (95% CI: 18.0–36.9) among TGW of all ages. Among participants aged 18–24 years, the prevalence of rectal CT ranged from 13.3% (4/30, 95% CI: 0.1–25.5) at baseline to 11.1% (3/27, 95% CI: 0.0–23.0) at 48 weeks (*p* = 0.80). Two (6.7%, 95% CI: 0.0–15.6) out of 30 young participants had rectal NG at baseline compared to 3 (11.1%, 95% CI: 0.0–23.0) of 27 at week 48 (*p* = 0.56). From baseline to 48 weeks, syphilis incidence was 36.6/100 person‐years (95% CI: 20.3–66.2) among TGW aged 18–24 years.

## 4 DISCUSSION

This study was the first PrEP demonstration study specific to TGW in Latin America. Despite the challenges of reaching and retaining this vulnerable population, our results showed high engagement in biomedical prevention research though adherence to PrEP decreased over time. The high PrEP retention among study participants is comparable to studies with MSM at 48 weeks of follow‐up, and higher than observed in studies with cisgender women sex workers and cisgender women who inject drugs [4, 5, 18, 19, 20]. Importantly, we found that social determinants of health significantly impacted study visit attendance and PrEP adherence. There was a significant decrease in study visit attendance and PrEP adherence over time, with youngest TGW having significantly higher odds of missed visits in our study. Adjusted models showed that low PrEP adherence was higher among TGW with less years of schooling and had an upward trend among Black participants, whereas receiving FHT from the study was associated with lower odds of low adherence. These results suggest that Brazilian TGW are interested and just as capable to adhere to oral medication to prevent HIV acquisition, but that social determinants of health must be addressed to improve adherence.

Despite the high PrEP retention, the proportion of TGW with protective drug concentrations decreased over the study follow‐up period, which is consistent with other studies. Lower drug concentration levels over time may be related to PrEP fatigue as long‐term adherence to daily medication is difficult to achieve in many contexts and across populations [21]. TGW enrolled in a qualitative study in the United States reported pill fatigue and frustration to add another medication to their daily regimens as a barrier to PrEP use [22]. Long‐acting (LA) PrEP agents are promising strategies to address adherence issues among vulnerable groups. Nevertheless, as many TGW use fillers in the gluteal area [6], this will limit their ability to use LA injectable cabotegravir as this must be injected into the gluteal muscle [23]. The use of fillers, usually referred to as industrial silicone, is extremely common in Brazil. In PrEParadas, 41.5% of participants had fillers, similar to previous Brazilian studies, which described a 32–49% prevalence among TGW, which is a far larger proportion than found in the United States (16.7%) [24].

Potential drug–drug interactions may also partially explain the lower TDF levels among TGW. Small pharmacokinetics studies described minor interactions between FHT and PrEP, but TDF and FTC concentrations remained above the threshold needed for protection and thus, clinically meaningful interactions are not expected [25]. Participants on the FHT available at the site had higher odds of PrEP adherence, which indicates that offering both HIV prevention and gender‐affirming care, including hormone provision, could facilitate PrEP implementation among TGW [8]. Peruvian TGW reported high interest in the integration of HIV services with FHT provision [26]. Among U.S. young TGW, those who received FHT were more willing to participate in a PrEP adherence study [27]. Even though offering trans‐specific care is not mandatory nor feasible in most settings, where possible, providing a gender‐affirmative approach may improve HIV care and prevention engagement among trans people [28].

Social determinants also emerged as important predictors of low adherence. TGW with less years of schooling had the lowest adherence. In addition, Black participants tended to have low PrEP adherence, which is consistent with other HIV research [5]. Among people living with HIV, lower schooling was associated with worse HIV care outcomes, such as late presentation to care [29], not being on antiretroviral treatment and not being virologically suppressed [30]. The combination of transphobic experiences, racism and discrimination poses Black TGW at a higher vulnerability to HIV and negatively impact their HIV care and prevention outcomes, including adherence [31, 32]. Black TGW and those with lower schooling may face considerable threats to their health and wellness, especially when they experience these factors together. The high prevalence and vicious interplay of these social determinants of health highlight the need to identify the specific barriers and evidence‐based interventions that will maximize PrEP adherence among TGW.

A positive finding from our study was the high PrEP retention. In a Peruvian study to support PrEP among TGW, about half of participants (49/89, 55%) were lost to follow‐up after 3 months [33]. Stigma and discrimination in the healthcare setting are critical barriers to HIV prevention for TGW. Prior discriminatory experiences in the healthcare setting represented an important impediment to accessing PrEP [34, 35, 36, 37]. Since 2015, our team has been building competency at serving TGW, implementing services to meet community needs and fostering connections with the trans community through research and outreach. In efforts to build a research site for trans‐specific studies, we developed a diverse group of researchers, including TGW who lent their expertise in planning and implementation of the PrEParadas study. We also provided gender‐affirming care as part of the study [38]. Our high PrEP retention is consistent with research stating that staff training on trans‐competency, trans‐friendly environments and partnerships with trans‐organizations could have positive implications for TGW's enrolment, retention and adherence in research studies [39]. Studies planning to focus on TGW could use the strategies we employed to maintain PrEP retention.

Although overall retention was high, age was a significant determinant of missing visits. Having a missed visit may be a proxy of individuals who were not using PrEP as prescribed since medications were delivered to cover the period until the next visit. In our study, TGW aged <25 years had higher rates of missed visits. Young age has been associated with worse PrEP outcomes, such as low awareness, uptake, retention and adherence [40]. Chan et al. observed that age <30 years was a significant predictor of not being retained in PrEP after 12 months of follow‐up [20]. In the PrEP Brasil study, both having young age and being TGW were associated with decreased odds of PrEP engagement (combining visit attendance and DBS levels) along the study [5]. Our group has previously described alarmingly high HIV prevalence among young Brazilian TGW aged 18–24 years (24.2%), along with lower odds of HIV prevention knowledge and higher odds of condomless sex regardless of HIV status compared to their older peers [41]. Yet, trans youth continue to be under‐represented in HIV research, including PrEP studies [27]. Interventions may be needed that address developmental needs related to access and utilization of PrEP among the youngest TGW.

Our study has some limitations. First, as our site is highly experienced in PrEP that also provides gender‐affirming care, our successful PrEP uptake and retention rates may not be generalizable to other settings. A proportion of eligible pre‐screened participants (141, 52%) did not enrol in this study, which may limit our ability to identify potential associations. Same‐day PrEP initiation, currently recommended in many settings, including ours, is an important approach to mitigate losses prior to PrEP engagement. Notwithstanding, PrEP retention remains an important issue. Our findings help to decrease the knowledge gaps related to PrEP retention among TGW. The current analysis does not include an exhaustive list of social determinants and future research addressing more robust explorations of social determinants is needed. In addition, our follow‐up time was 1 year, and results may differ in longer periods. Also, we did not perform a power calculation, which may have reduced our ability to detect statistical significance in the evaluation of potential associations. Sample limitations may have hindered to find a significant association between PrEP adherence and race. We only screened CT/NG twice during the follow‐up since this screening is not available routinely in Brazil. Finally, our data collection occurred prior to COVID‐19 epidemic, which will definitely change the HIV prevention scenario.

## 5 CONCLUSIONS

High PrEP retention can be achieved in PrEP programs with TGW when implemented in a gender‐affirming setting. PrEP adherence may be an important challenge faced among TGW due to racial and socio‐economic disparities. HIV prevention trials and the scale up of known efficacious prevention tools like PrEP will have to address systemic social determinants TGW face that are unlike most other populations at risk of acquiring HIV.

## COMPETING INTERESTS

PA has received consulting fees from Gilead, Merck and ViiV, and research funding paid to his institution from Gilead. All other authors declare no competing interests.

## AUTHORS’ CONTRIBUTIONS

EMJ, BH, SWC, VGV and BG conceived the study. EMJ, TST, PML, EW and BG interpreted the findings and drafted the manuscript. ICL and MC did the statistical analyses with aid from RIM, EMJ and PML. Rita de Cassia Elias Estrela (RCEE), MR, LM, CRVC and PML helped with data acquisition, interpretation of the findings and drafting the manuscript. SWC, VGV and PA were involved in revising the manuscript for important intellectual content. All authors read and approved the final manuscript.

## PREPARADAS STUDY TEAM

Isabele Moura, Leonardo Eksterman, Daniel M McMahon Waite, Desirée Vieira, José Roberto Granjeiro, Josias Freitas, Toni Santos, Nilo Fernandes, Vitoria Berg Cattani, Sandro Nazer, Luana M. S. Marins, Valéria R. T. Ribeiro, Robson P. N. Silva, Giovanna G. Costa, Ana Carolina Vieira, Renata A. Bastos, Aline Alves, Tania Krstic, Ana Cristina G. Ferreira, Monica Derrico, Luciana Kamel, Cristina M. Jalil, Eduardo Carvalheira Netto, Marcos Davi G. de Sousa, Pedro Leite, Kim Geraldo Mattos, Jessica Bezerra Felix, Tamires Vilela Baião, Gisele Hottz, Natália Gomes Maia, Tamiris Paixão da Silva, Tiago Porto.

## FUNDING

This study was sponsored by the Brazilian Ministry of Health (Brasília, Brazil; #01/2013 BRA/K57) and Secretaria de Vigilancia em Saúde (SVS; #281/2013). Gilead Sciences donated the study drug and covered costs related to drug concentration assessment, but had no role in study design, collection, analysis and interpretation of data, writing of the manuscript or the decision to submit the manuscript for publication.

## Data Availability

Data is not available via a publicly available data repository.
